# Air and Noise Pollution Exposure in Early Life and Mental Health From Adolescence to Young Adulthood

**DOI:** 10.1001/jamanetworkopen.2024.12169

**Published:** 2024-05-28

**Authors:** Joanne B. Newbury, Jon Heron, James B. Kirkbride, Helen L. Fisher, Ioannis Bakolis, Andy Boyd, Richard Thomas, Stanley Zammit

**Affiliations:** 1 Population Health Sciences, Bristol Medical School, University of Bristol, Bristol, United Kingdom; 2 Social, Genetic, and Developmental Psychiatry Centre, Institute of Psychiatry, Psychology & Neuroscience, King’s College London, London, United Kingdom; 3 PsyLife Group, Division of Psychiatry, University College London, London, United Kingdom; 4 ESRC Centre for Society and Mental Health, King’s College London, London, United Kingdom; 5 Department of Biostatistics and Health Informatics, Institute of Psychiatry, Psychology & Neuroscience, King’s College London, London, United Kingdom; 6 Centre for Implementation Science, Health Service and Population Research Department, Institute of Psychiatry, Psychology & Neuroscience, King’s College London, London, United Kingdom; 7 UK Longitudinal Linkage Collaboration, University of Bristol, Bristol, United Kingdom; 8 MRC Centre for Neuropsychiatric Genetics and Genomics, School of Medicine, Cardiff University, Cardiff, United Kingdom

## Abstract

**Importance:**

Growing evidence associates air pollution exposure with various psychiatric disorders. However, the importance of early-life (eg, prenatal) air pollution exposure to mental health during youth is poorly understood, and few longitudinal studies have investigated the association of noise pollution with youth mental health.

**Objectives:**

To examine the longitudinal associations of air and noise pollution exposure in pregnancy, childhood, and adolescence with psychotic experiences, depression, and anxiety in youths from ages 13 to 24 years.

**Design, Setting, and Participants:**

This cohort study used data from the Avon Longitudinal Study of Parents and Children, an ongoing longitudinal birth cohort founded in 1991 through 1993 in Southwest England, United Kingdom. The cohort includes over 14 000 infants with due dates between April 1, 1991, and December 31, 1992, who were subsequently followed up into adulthood. Data were analyzed October 29, 2021, to March 11, 2024.

**Exposures:**

A novel linkage (completed in 2020) was performed to link high-resolution (100 m^2^) estimates of nitrogen dioxide (NO_2_), fine particulate matter under 2.5 μm (PM_2.5_), and noise pollution to home addresses from pregnancy to 12 years of age.

**Main outcomes and measures:**

Psychotic experiences, depression, and anxiety were measured at ages 13, 18, and 24 years. Logistic regression models controlled for key individual-, family-, and area-level confounders.

**Results:**

This cohort study included 9065 participants who had any mental health data, of whom (with sample size varying by parameter) 51.4% (4657 of 9051) were female, 19.5% (1544 of 7910) reported psychotic experiences, 11.4% (947 of 8344) reported depression, and 9.7% (811 of 8398) reported anxiety. Mean (SD) age at follow-up was 24.5 (0.8) years. After covariate adjustment, IQR increases (0.72 μg/m^3^) in PM_2.5_ levels during pregnancy (adjusted odds ratio [AOR], 1.11 [95% CI, 1.04-1.19]; *P* = .002) and during childhood (AOR, 1.09 [95% CI, 1.00-1.10]; *P* = .04) were associated with elevated odds for psychotic experiences. Pregnancy PM_2.5_ exposure was also associated with depression (AOR, 1.10 [95% CI, 1.02-1.18]; *P* = .01). Higher noise pollution exposure in childhood (AOR, 1.19 [95% CI, 1.03-1.38]; *P* = .02) and adolescence (AOR, 1.22 [95% CI, 1.02-1.45]; *P* = .03) was associated with elevated odds for anxiety.

**Conclusions and Relevance:**

In this longitudinal cohort study, early-life air and noise pollution exposure were prospectively associated with 3 common mental health problems from adolescence to young adulthood. There was a degree of specificity in terms of pollutant-timing-outcome associations. Interventions to reduce air and noise pollution exposure (eg, clean air zones) could potentially improve population mental health. Replication using quasi-experimental designs is now needed to shed further light on the underlying causes of these associations.

## Key Points

**Question:**

Is exposure to air and noise pollution in pregnancy, childhood, and adolescence associated with the development of psychotic experiences, depression, and anxiety between 13 and 24 years of age?

**Findings:**

In this longitudinal birth cohort study followed up into adulthood that included 9065 participants with mental health data, higher exposure to fine particulate matter (PM_2.5_) in pregnancy and childhood was associated with increased psychotic experiences and in pregnancy was associated with higher rates of depression. Higher noise pollution exposure in childhood and adolescence was associated with increased anxiety.

**Meaning:**

These findings build on evidence associating air and noise pollution with mental health, highlighting a role of early-life pollution exposure in youth mental health problems.

## Teaser

This cohort study investigates the associations between air and noise pollution exposure in pregnancy through age 12 years with the development of psychotic experiences, depression, and anxiety at 13 to 24 years of age.

## Introduction

Childhood, adolescence, and early adulthood are critical periods for the development of psychiatric disorders: worldwide, nearly two-thirds of individuals affected become unwell by 25 years of age.^1^ Identifying early-life risk factors is a crucial research challenge in developing preventative interventions and improving lifelong mental health trajectories.

Growing evidence suggests that air pollution exposure may be associated with the onset of psychiatric problems, including mood, affective, and psychotic disorders.^2,3,4,5,6^ Air pollution comprises toxic gases and particulate matter (ie, organic and inorganic solid and liquid aerosols) of mostly anthropogenic origin.^7^ Understanding the potential effect of air pollution on mental health is increasingly crucial, given the human and societal cost of poor mental health,^8^ the global shift toward urban living,^9,10^ and the backdrop of emissions-induced climate change.^11^ Air pollution could negatively affect mental health via numerous pathways, including by compromising the blood-brain barrier, promoting neuroinflammation and oxidative stress, and directly entering the brain and damaging tissue therein.^12,13^ However, key research gaps remain. First, the relative importance of early-life exposure, including prenatal exposure, is uncertain. Infants and children are thought to be especially vulnerable to air pollution,^14,15^ but longitudinal, high-resolution pollution data spanning the early years of human life are scarce. Second, relatively few studies have examined the association of air pollution with youth mental health problems,^16^ despite youth being a critical period for intervention. Third, few longitudinal studies have investigated the role of noise pollution in mental health,^17^ despite the correlation between noise and air pollution.^18^ Finally, studies have often used crude pollution data and lacked adequate controls for potential confounders.

We aimed to advance understanding on this topic by capitalizing on a novel linkage between high-resolution outdoor air and noise pollution data and a cohort of over 14 000 infants born in Southwest England in 1991 through 1993 and followed up into adulthood. We examined the association of air and noise pollution exposure from pregnancy to 12 years of age with mental health problems from ages 13 to 24 years. Based on previous evidence, we focused on psychotic experiences (eg, subclinical hallucinations and delusions), depression, and anxiety. These problems are common^1,19,20,21^ and increasing^22^ among youth and strongly predict future psychopathology,^23,24^ making them useful and important targets. We hypothesized that participants exposed to higher air and noise pollution would subsequently experience worse mental health.

## Methods

### Participants

The Avon Longitudinal Study of Parents and Children (ALSPAC) is a UK birth cohort,^25,26,27,28^ described further in the eMethods in Supplement 1. Briefly, pregnant women residing in and around the City of Bristol (population approximately 714 000 in 2024) in Southwest England with due dates between April 1, 1991, and December 31, 1992, were approached to take part in the study. The initial number of pregnancies enrolled was 14 551, resulting in 13 988 children alive at 1 year of age. At age 7 years, the initial sample was bolstered with additional eligible cases, resulting in 14 901 infants alive at 1 year of age. The catchment area has a mix of urban, suburban, and rural environments.^29^ The study website contains details of all the data and a fully searchable data dictionary and variable search tool.^30^ Ethical approval for the study was obtained from the ALSPAC Ethics and Law Committee and the Local Research Ethics Committees. Informed consent for the use of data collected via questionnaires and clinics was obtained from participants following the recommendations of the ALSPAC Ethics and Law Committee at the time. The present study is reported according to the Strengthening the Reporting of Observational Studies in Epidemiology (STROBE) reporting guideline.^31^

### Psychotic Experiences

Psychotic experiences were measured at ages 13, 18, and 24 years using a semi-structured interview^32^ that consisted of 12 core items about hallucinations, delusions, and thought interference, rated against the Schedule for Clinical Assessment in Neuropsychiatry version 2.0 (SCAN 2.0).^33^ Consistent with previous ALSPAC studies,^34,35^ psychotic experiences were defined such that 0 represented none, and 1 represented suspected or definite. The reporting period at each phase was since the participant’s 12th birthday. At 13 years of age, 13.6% (926 of 6788) of participants reported psychotic experiences, at 18 years of age 9.2% (432 of 4715) reported psychotic experiences, and at 24 years of age, 12.6% (491 of 3888) reported psychotic experiences. We summed psychotic experiences across time points and dichotomized the variable for analyses such that participants received a score of 1 for suspected or definite psychotic experiences if they reported psychotic experiences at any age.

### Depression and Anxiety

Depression and anxiety were measured at age 13 years via parent-completed Development and Well-being Assessments.^36^ Responses were classified into probabilistic bands according to *Diagnostic and Statistical Manual of Mental Disorders (Fourth Edition)* criteria for major depressive disorder and generalized anxiety disorder, and dichotomized for analysis (bands 0-2, 0; bands 3-5, 1). At ages 18 and 24 years, depression and anxiety were measured using the Clinical Interview Schedule Revised,^37^ a self-administered computerized interview that gave *International Statistical Classification of Diseases, Tenth Revision*, diagnoses of moderate to severe depression and generalized anxiety disorder. The reporting period at each phase was the past month, although a 6-month reporting period was used for anxiety at 13 years of age. At 13 years of age, 5.6% (386 of 6944 of participants) reported depression and 3.6% (254 of 7044) reported anxiety. At 18 years of age, 7.9% (359 of 4560) reported depression and 5.7% (262 of 4560) reported anxiety. At 24 years of age, 7.7% (304 of 3965) reported depression and 9.8% (386 of 3956) reported anxiety. We summed depression and anxiety across time points and dichotomized the variables for analysis such that participants received a score of 1 if they had depression or anxiety at any age.

### Air Pollution

Air pollutants included nitrogen dioxide (NO_2_) and fine particulate matter with a diameter smaller than 2.5 μm (PM_2.5_). Both pollutants have well-established health impacts^10^ and more recent associations with psychiatric disorders.^5^ These air pollutants were estimated as part of the LifeCycle project^38^ using the Effects of Low-Level Air Pollution: A Study in Europe (ELAPSE) model, which is described elsewhere and further in the eMethods in Supplement 1.^39^ Briefly, the ELAPSE model is a hybrid land-use regression model for Europe that derived concentrations of NO_2_ and PM_2.5_ in 2010. The model produces annualized estimates at 100 m^2^ resolution, explaining 59% and 71% of measured spatial variability for NO_2_ and PM_2.5_, respectively.^39^ Estimates were linked to residential geocodes from pregnancy to age 12 years for participants who had lived in the original ALSPAC catchment area^29^ up to 12 years of age and provided permission for geospatial linkage. Linkage was completed in 2020.

### Noise Pollution

Residential noise pollution exposure was also estimated as part of the LifeCycle project^38^ based on the UK Government’s Department for Environment, Food and Rural Affairs 2006 road traffic noise map. Data represent an annualized mean of day and night noise pollution, categorized according to low to medium (<55 dB: the European Environment Agency’s threshold^40^), high (55-60 dB), and very high (>60 dB) noise. eFigure 1 in Supplement 1 shows the correlation between noise pollution, NO_2_, and PM_2.5_ across time points.

### Covariates

Potential confounders were informed by the literature and formally selected using a directed acyclic graph (eFigure 2 in Supplement 1). We considered individual- and family-level covariates that could be associated with mental health problems and with downward mobility into more polluted neighborhoods. These included ethnicity self-reported by mothers during pregnancy, family psychiatric history, maternal social class, maternal education, and housing tenure. Area-level covariates included population density, neighborhood deprivation, social fragmentation, and greenspace and were time varying, corresponding to the timing of pollution exposure. Covariates are described fully in the eMethods in Supplement 1 and briefly below.

#### Individual- and Family-Level Covariates

Race and ethnic group was reported by mothers during pregnancy, with specific categories to select including Bangladeshi, Black/African, Black/Caribbean, Black/other, Chinese, Indian, Pakistani, White, and any other ethnic group. Family psychiatric problems were reported by mothers and fathers during pregnancy and defined as the presence of any psychiatric problem affecting the mother, father, or any biological grandparent. Maternal social class based on occupation was reported by mothers during pregnancy. Maternal education was reported by mothers when infants were around 8 months. Home ownership was reported by mothers during pregnancy.

#### Neighborhood-Level Covariates

Population density was derived from 1991 and 2001 census data.^35^ Area-level deprivation was based on the Index of Multiple Deprivation 2000.^41^ Social fragmentation was based on a z-scored sum of census data on residential mobility, marital status, single-person households, and home ownership.^35^ Greenspace was assessed based on the Normalized Difference Vegetation Index.^42^

### Statistical Analysis

Analyses were performed from October 29, 2021, to March 11, 2024, in Stata, version 18.0 (StataCorp LLC). The code can be found at GitHub.^43^ The characteristics of the sample with vs without mental health data were described according to percentages, means, and standard deviations. Group differences were explored using χ^2^ and *t* tests. To explore the importance of different exposure periods, we derived exposure estimates for 3 developmental stages, pregnancy, childhood (birth to age 9 years), and adolescence (ages 10-12 years),^44^ which were calculated using mean exposure values for NO_2_, PM_2.5_, and noise pollution during these age windows. Given that NO_2_ and PM_2.5_ had very different absolute ranges, scores were standardized by dividing by the IQR. To aid comparison between air and noise pollution, we treated noise pollution as a continuous variable, assuming a normal distribution underlying the categorical variable. Results treating noise as categorical are reported in eTable 1 in Supplement 1.

For main analyses, logistic regression was used to examine the associations of NO_2_, PM_2.5_, and noise pollution in pregnancy, childhood, and adolescence with the mental health outcomes. We conducted an unadjusted model (model 1), then adjusted for individual- and family-level covariates (model 2), and then additionally adjusted for area-level covariates (model 3). To better understand the independent associations from different exposure periods, we then adjusted childhood and adolescent exposure for previous exposure (model 4). However, given that the high correlation between pollutants over time (eFigure 1 in Supplement 1) could introduce multicollinearity, we interpreted model 4 with caution. To estimate residual confounding, we also calculated E values^45^ for models 3 and 4, which indicate the strength of association that an unmeasured confounder would require to nullify associations. All models accounted for potential hierarchy in the data by clustering around the lower layer super output area (containing a mean of about 1500 residents) using the cluster command, which provides robust SEs adjusted for within cluster correlated data.^46^ All analyses were conducted following multiple imputation by chained equations,^47^ described in the eMethods in Supplement 1. A 2-sided value of *P* < .05 was considered statistically significant.

We conducted 3 sensitivity analyses. First, we analyzed NO_2_, PM_2.5_, and noise pollution simultaneously, to control each for the others and address potential copollutant confounding. Second, we restricted analyses to participants who did not move house from pregnancy to age 12 years (29.8%) to keep pollution levels as consistent as possible over time. Third, we repeated main analyses for individuals with complete data.

## Results

### Sample Characteristics

The study included 9065 participants (mean [SD] age at follow-up, 24.5 [0.8] years) who had any mental health data, of whom (with sample sizes varying by parameter) 51.4% (4657 of 9051) were female, 48.6% (4394 of 9051) were male, 95.8% (7616 of 7954) were ethnically White, and 4.2% (338 of 7954) were of other ethnicity (which included Bangladeshi, Black African, Black Caribbean, Chinese, Indian, Pakistani, and others; these categories were collapsed into one because numbers in some categories were small enough to increase the risk of identification). In addition, 19.5% (1544 of 7910) reported psychotic experiences, 11.4% (947 of 8344) reported depression, and 9.7% (811 of 8398) reported anxiety (Table 1). Over half of participants (60.8% [4793 of 7886]) had a family psychiatric history; 21.8% (1583 of 7248) had mothers who worked in manual occupations; 15.7% (1274 of 8093) had mothers with degrees; and 81.6% (6670 of 8176) lived in homes owned by their parent (or parents). Mean (SD) population density was 33 (21) persons per hectare, and 19.3% (933 of 4831) of participants lived in the most deprived neighborhoods. The sample with vs without mental health data differed for most variables: participants with mental health data were more likely to be female, be White, have a family psychiatric history, and have more advantaged characteristics across the other variables. These differences should be borne in mind when interpreting the results.

**Table 1.** Sample Characteristics for Participants With vs Without Mental Health Data

Sample characteristics^a^	Participants, No. (%) (N = 9065)		χ^2^ or *t*	*P* value
	Sample with mental health data	Sample without mental health data		
Psychotic experiences (ages 13-24 y) (n = 7910)				
No	6366 (80.5)	NA	NA	NA
Yes	1544 (19.5)	NA	NA	NA
Depression (ages 13-24 y) (n = 8344)			NA	NA
No	7397 (88.7)	NA	NA	NA
Yes	947 (11.4)	NA	NA	NA
Anxiety (ages 13-24 y) (n = 8398)			NA	NA
No	7587 (90.3)	NA	NA	NA
Yes	811 (9.7)	NA	NA	NA
Sex (n = 9051)				
Female	4657 (51.4)	2691 (45.0)	60.9	<.001
Male	4394 (48.6)	3295 (55.0)		
Ethnicity (n = 7954)				
White	7616 (95.8)	3906 (93.4)	31.0	<.001
All other ethnicities^b^	338 (4.2)	275 (6.6)		
Family psychiatric history (n = 7886)				
No	3093 (39.2)	2569 (80.8)	1600.0	<.001
Yes	4793 (60.8)	610 (19.2)		
Maternal social class (n = 7248)^c^				
Professional	295 (4.1)	73 (1.9)	258.3	<.001
Managerial and technical	2302 (31.8)	849 (22.0)		
Skilled nonmanual	3068 (42.3)	1656 (42.9)		
Skilled manual	264 (3.6)	188 (4.9)		
Partly skilled	1096 (15.1)	867 (22.4)		
Unskilled	223 (3.1)	230 (6.0)		
Maternal education (n = 8093)				
Degree	1274 (15.7)	334 (7.6)	693.1	<.001
A level	2087 (25.8)	706 (16.1)		
O level	2850 (35.2)	1472 (33.6)		
Vocational	730 (9.0)	499 (11.4)		
CSE	1152 (14.2)	1373 (31.3)		
House tenure (n = 8176)				
Mortgaged or owned	6670 (81.6)	3200 (60.3)	744.6	<.001
Rented	1506 (18.4)	2109 (39.7)		
Population density, mean (SD) (n = 7438)^d^	33 (21)	35 (19)	4.3	<.001
Area-level deprivation (n = 4831)				
1 (Least deprived)	1419 (29.4)	596 (19.7)	179.9	<.001
2	830 (17.2)	456 (15.0)		
3	785 (16.3)	515 (17.0)		
4	864 (17.9)	529 (17.4)		
5 (Most deprived)	933 (19.3)	937 (30.9)		
Social fragmentation (n = 7437)^e^	−0.28 (2.9)	−0.11 (2.8)	2.9	.003
Greenspace (n = 7437)^f^	0.41 (0.1)	0.42 (0.1)	−3.4	<.001
NO_2_, mean (SD), μm/m^3^ (n = 7404)	26.93 (4.2)	27.08 (4.0)	2.0	.047
PM_2.5_, mean (SD), μm/m^3^ (n = 7404)	13.32 (0.9)	13.38 (0.8)	3.9	<.001
Noise pollution (n = 5221)				
Low (<55 dB)	1594 (930.5)	1010 (30.1)	3.1	.213
Medium (55-60 dB)	2442 (46.8)	1531 (45.6)		
High (>60 dB)	1185 (22.7)	817 (24.3)		

Abbreviations: CSE, certificate of secondary education; NA, not applicable; NO_2_, nitrogen dioxide; PM_2.5_, particulate matter under 2.5 μm/m^3^.

^a^ Sample sizes for some parameters varied.

^b^ Due to small numbers of participants, all races and ethnic groups other than White were grouped. These races and ethnicities included Bangladeshi, Black African, Black Caribbean, Chinese, Indian, Pakistani, and other ethnicities.

^c^ Based on maternal occupation.

^d^ Unit is persons per hectare.

^e^ Sum of *z*-scored census information on population turnover, unmarried people, single-person households, and privately rented households.

^f^ Unit is the normalized difference vegetation index: range −1 to 1.

### Air and Noise Pollution Exposure

**Figure 1.**
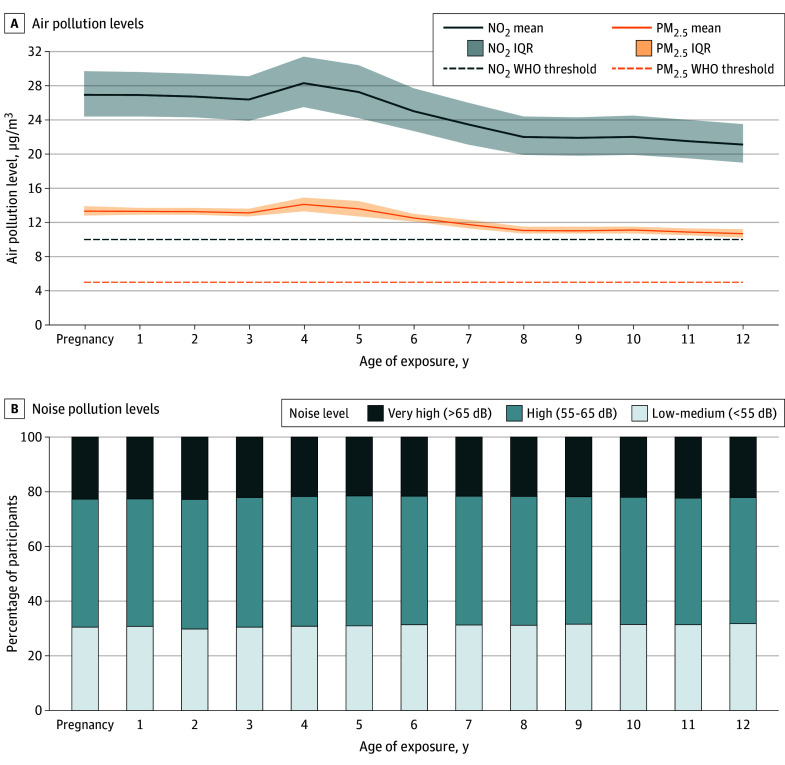
Air and Noise Pollution Exposure From Pregnancy to 12 Years of Age in the Avon Longitudinal Study of Parents and Children Study Sample NO_2_ indicates nitrogen dioxide; PM_2.5_, particulate matter under 2.5 μm; and WHO, World Health Organization. Shading in panel A represents IQRs.

Figure 1A shows estimated levels of NO_2_ and PM_2.5_ for the sample, alongside the World Health Organization’s (WHO) 2021 exposure thresholds.^48^ Mean (SD) levels of NO_2_ (eg, 26.9 [4.2] μg/m^3^ in pregnancy vs 21.1 [3.5] μg/m^3^ at 12 years of age) and PM_2.5_ (eg, 13.3 [0.9] μg/m^3^ in pregnancy vs 10.7 [0.8] μg/m^3^ at 12 years of age) decreased slightly over time. However, the mean exposure at age 12 years remained above the WHO’s thresholds for both pollutants (NO_2_, 10.0 μg/m^3^; PM_2.5_, 5.0 μg/m^3^). Additionally, over two-thirds of participants were exposed to high or very high noise pollution,^40^ which changed little over time (eg, 22.7% in pregnancy vs 22.2% at year 12 for high noise pollution) (Figure 1B).

### Associations of Air and Noise Pollution With Mental Health

**Figure 2.**
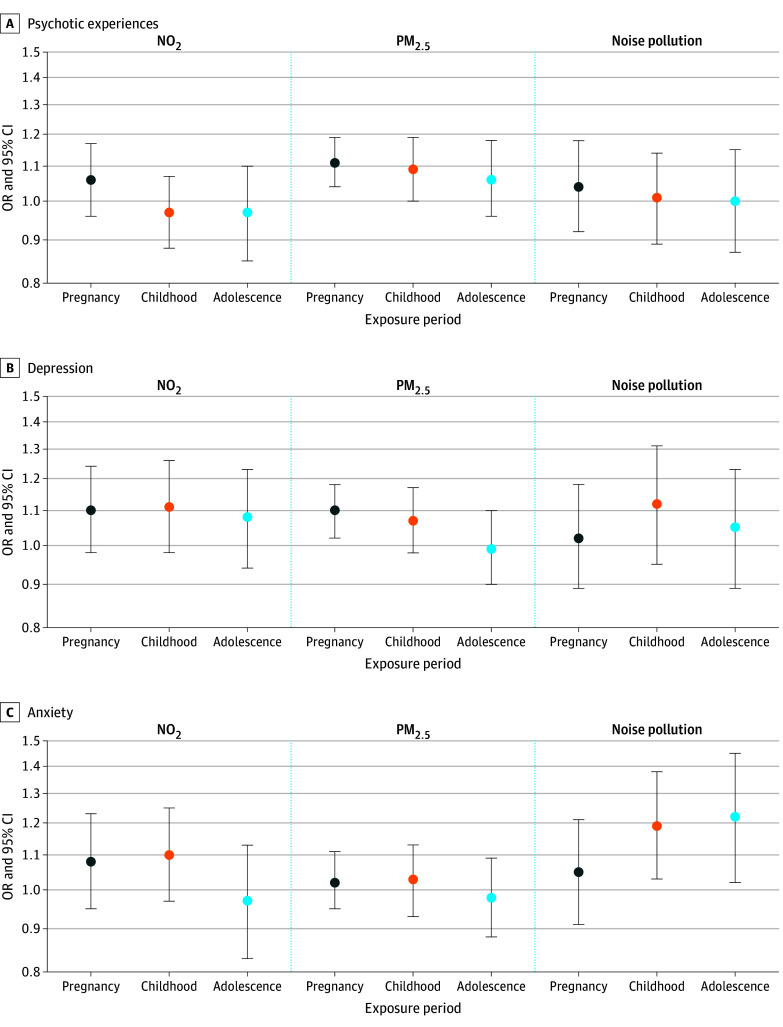
Adjusted Associations of Early-Life Air and Noise Pollution Exposure With Youth Mental Health Problems Results are from model 3, which is adjusted for ethnicity, family psychiatric history, maternal social class, maternal education, house tenure, population density, neighborhood deprivation, social fragmentation, and greenspace. Sample sizes of imputed data sets range from 2952 (adolescence noise pollution and psychotic experiences) to 6154 (pregnancy air pollution and anxiety). NO_2_ indicates nitrogen dioxide; OR, odds ratio; and PM_2.5_, particulate matter less than 2.5 μm.

Associations of levels of NO_2,_ PM_2.5_, and noise pollution with psychotic experiences, depression, and anxiety are given in Table 2, which shows unadjusted and adjusted results alongside E values, and Figure 2, which shows model 3 results. Before covariate adjustment, IQR (4.47 μg/m^3^) increases in NO_2_ levels during pregnancy were associated with elevated odds for psychotic experiences (odds ratio [OR], 1.08, [95% CI, 1.00-1.17]; *P* = .04). However, there was no association after adjusting for area-level covariates. In contrast, following covariate adjustment, IQR (0.72 μg/m^3^) increases in PM_2.5_ during pregnancy (adjusted [A]OR, 1.11 [95% CI, 1.04-1.19]; *P* = .002) and childhood (AOR, 1.09 [95% CI, 1.00-1.19]; *P* = .04) were associated with elevated odds for psychotic experiences, although for childhood exposure (model 4), there was no association after adjusting for pregnancy exposure. There was no association between noise pollution and psychotic experiences (eg, AOR, 1.04 [95% CI, 0.92-1.18]; *P* = .50 during pregnancy).

**Table 2.** Associations of Early-Life Air and Noise Pollution Exposure With Youth Mental Health Problems^a^

Outcome	Pregnancy exposure			Childhood exposure			Adolescence exposure		
Pollutant and model	OR (95% CI)	*P* value	E value OR (LCL)^b^	OR (95% CI)	*P* value	E value OR (LCL)^b^	OR (95% CI)	*P* value	E value OR (LCL)^b^
**Psychotic experiences**									
NO_2_									
Model 1^c^	1.08 (1.00-1.17)	.04	NA	1.05 (0.97-1.14)	.24	NA	1.06 (0.96-1.17)	.28	NA
Model 2^d^	1.08 (1.00-1.17)	.05	NA	1.04 (0.96-1.13)	.32	NA	1.04 (0.95-1.16)	.39	NA
Model 3^e^	1.06 (0.96-1.17)	.28	1.31 (1.00)	0.97 (0.88-1.07)	.55	1.21 (1.00)	0.97 (0.85-1.10)	.58	1.21 (1.00)
Model 4^f^	NA	NA	NA	0.89 (0.77-1.03)	.11	1.50 (1.00)	1.02 (0.81-1.28)	.89	1.16 (1.00)
PM_2.5_									
Model 1^c^	1.11 (1.04-1.18)	.001	NA	1.11 (1.03-1.19)	.009	NA	1.09 (0.99-1.21)	.07	NA
Model 2^d^	1.11 (1.04-1.18)	.001	NA	1.10 (1.02-1.19)	.01	NA	1.09 (0.98-1.20)	.10	NA
Model 3^e^	1.11 (1.04-1.19)	.002	1.46 (1.24)	1.09 (1.00-1.19)	.04	1.40 (1.00)	1.06 (0.96-1.18)	.25	1.31 (1.00)
Model 4^f^	NA	NA	NA	1.00 (0.90-1.12)	.93	1.00 (1.00)	1.02 (0.84-1.24)	.82	1.16 (1.00)
Noise									
Model 1^c^	1.06 (0.94-1.20)	.36	NA	1.04 (0.92-1.17)	.57	NA	1.01 (0.89-1.15)	.85	NA
Model 2^d^	1.06 (0.93-1.20)	.38	NA	1.03 (0.91-1.17)	.62	NA	1.00 (0.87-1.14)	.98	NA
Model 3^e^	1.04 (0.92-1.18)	.50	1.24 (1.00)	1.01 (0.89-1.14)	.88	1.11 (1.00)	1.00 (0.87-1.15)	.99	1.00 (1.00)
Model 4^f^	NA	NA	NA	0.95 (0.79-1.15)	.62	1.29 (1.00)	0.99 (0.81-1.21)	.90	1.11 (1.00)
**Depression**									
NO_2_									
Model 1^c^	1.06 (0.97-1.15)	.19	NA	1.09 (0.99-1.20)	.09	NA	1.09 (0.98-1.22)	.12	NA
Model 2^d^	1.06 (0.97-1.15)	.19	NA	1.08 (0.98-1.19)	.12	NA	1.08 (0.97-1.20)	.18	NA
Model 3^e^	1.10 (0.98-1.24)	.10	1.43 (1.00)	1.11 (0.98-1.26)	.09	1.46 (1.00)	1.08 (0.94-1.23)	.28	1.37 (1.00)
Model 4^f^	NA	NA	NA	1.09 (0.89-1.33)	.42	1.40 (1.00)	0.96 (0.72-1.28)	.77	1.25 (1.00)
PM_2.5_									
Model 1^c^	1.07 (1.00-1.15)	.04	NA	1.06 (0.97-1.14)	.18	NA	1.02 (0.93-1.12)	.66	NA
Model 2^d^	1.07 (1.00-1.15)	.04	NA	1.05 (0.97-1.14)	.25	NA	1.01 (0.92-1.11)	.82	NA
Model 3^e^	1.10 (1.02-1.18)	.01	1.43 (1.16)	1.07 (0.98-1.17)	.15	1.34 (1.00)	0.99 (0.90-1.10)	.90	1.11 (1.00)
Model 4^f^	NA	NA	NA	0.97 (0.86-1.11)	.69	1.21 (1.00)	0.89 (0.71-1.13)	.36	1.50 (1.00)
Noise									
Model 1^c^	1.03 (0.90-1.19)	.66	NA	1.13 (0.97-1.31)	.12	NA	1.08 (0.92-1.26)	.35	NA
Model 2^d^	1.03 (0.90-1.18)	.69	NA	1.12 (0.96-1.30)	.15	NA	1.07 (0.91-1.25)	.41	NA
Model 3^e^	1.02 (0.89-1.18)	.74	1.16 (1.00)	1.12 (0.95-1.31)	.17	1.49 (1.00)	1.05 (0.89-1.23)	.58	1.28 (1.00)
Model 4^f^	NA	NA	NA	1.20 (0.97-1.49)	.09	1.69 (1.00)	1.06 (0.80-1.40)	.68	1.31 (1.00)
**Anxiety**									
NO_2_									
Model 1^c^	1.14 (1.04-1.26)	.006	NA	1.15 (1.03-1.27)	.009	NA	1.05 (0.93-1.19)	.40	NA
Model 2^d^	1.14 (1.04-1.26)	.007	NA	1.14 (1.03-1.27)	.01	NA	1.05 (0.93-1.19)	.40	NA
Model 3^e^	1.08 (0.95-1.23)	.27	1.37 (1.00)	1.10 (0.97-1.25)	.15	1.43 (1.00)	0.97 (0.83-1.13)	.73	1.21 (1.00)
Model 4^f^	NA	NA	NA	0.97 (0.79-1.21)	.81	1.21 (1.00)	0.77 (0.57-1.03)	.08	1.92 (1.00)
PM_2.5_									
Model 1^c^	1.04 (0.97-1.12)	.22	NA	1.04 (0.96-1.13)	.34	NA	1.00 (0.91-1.10)	.98	NA
Model 2^d^	1.05 (0.98-1.12)	.19	NA	1.05 (0.96-1.14)	.30	NA	1.01 (0.91-1.11)	.90	NA
Model 3^e^	1.02 (0.95-1.11)	.55	1.16 (1.00)	1.03 (0.93-1.13)	.58	1.21 (1.00)	0.98 (0.88-1.09)	.69	1.16 (1.00)
Model 4^f^	NA	NA	NA	0.97 (0.84-1.12)	.67	1.21 (1.00)	0.95 (0.75-1.20)	.65	1.29 (1.00)
Noise									
Model 1^c^	1.01 (0.88-1.16)	.86	NA	1.13 (0.98-1.31)	.09	NA	1.17 (0.98-1.39)	.08	NA
Model 2^d^	1.03 (0.90-1.18)	.67	NA	1.15 (1.00-1.33)	.06	NA	1.19 (1.00-1.43)	.05	NA
Model 3^e^	1.05 (0.91-1.21)	.51	1.28 (1.00)	1.19 (1.03-1.38)	.02	1.67 (1.21)	1.22 (1.02-1.45)	.03	1.74 (1.16)
Model 4^f^	NA	NA	NA	1.32 (1.04-1.68)	.02	1.97 (1.24)	0.94 (0.68-1.29)	.71	1.32 (1.00)

Abbreviations: LCL, lower confidence limit; NA, not applicable; NO_2_, nitrogen dioxide; OR, odds ratio; PM_2.5_, particulate matter under 2.5 μm.

^a^ Sample size range, 2962 (adolescence noise pollution and psychotic experiences) to 6180 (pregnancy air pollution and anxiety).

^b^ The E values do not include upper confidence limits or *P* values.

^c^ Unadjusted.

^d^ Adjusted for individual- and family-level covariates.

^e^ Additionally adjusted for area-level covariates.

^f^ Additionally adjusted for earlier exposure. We interpret model 4 with caution given that high correlations across time points could lead to multicollinearity.

Following covariate adjustment, IQR increases in PM_2.5_ during pregnancy were associated with elevated odds for depression (eg, AOR, 1.10 [95% CI, 1.02-1.18]; *P* = .01 during pregnancy). There were no associations between NO_2_ (eg, AOR, 1.10 [95% CI, 0.98-1.24]; *P* = .10 during pregnancy) or noise pollution (eg, AOR, 1.02 [95% CI, 0.89-1.18]; *P* = .74 during pregnancy) and depression.

Before covariate adjustment, IQR increases in NO_2_ in pregnancy (OR, 1.14 [95% CI, 1.04-1.26]; *P* = .006) and childhood (OR, 1.15 [95% CI, 1.03-1.27]; *P* = .009) were associated with elevated odds for anxiety, but associations were attenuated to the null after adjusting for area-level covariates. There were no associations between PM_2.5_ exposure during childhood and anxiety (AOR, 1.10 [95% CI, 0.97-1.25]; *P* = .58 for model 3). In contrast, participants exposed to higher noise pollution in childhood (AOR, 1.19 [95% CI, 1.03-1.38]; *P* = .02) and in adolescence (AOR, 1.22 [95% CI, 1.02-1.45]; *P* = .03) had elevated odds for anxiety; however, adolescent exposure was attenuated to the null after controlling for pregnancy and childhood exposure (model 4). eTable 1 in Supplement 1 gives results when noise pollution was treated as categorical. This analysis highlighted several dose-response associations, although no difference in model fit was observed compared with the main results.

### E Values

In eTables 2 and 3 in Supplement 1, we take as examples the associations of pregnancy PM_2.5_ with psychotic experiences and adolescent noise pollution with anxiety from model 3 and compare the E values to the associations from included covariates. The E value ORs were 1.46 (lower confidence limit, 1.24) for pregnancy PM_2.5_ with psychotic experiences and 1.74 (lower confidence limit, 1.16) for adolescent noise pollution with anxiety. These E value ORs were larger in magnitude than the ORs for associations of the covariates with the exposures and outcomes, indicating that an unmeasured confounder would require a relatively strong confounding influence to nullify associations.

### Sensitivity Analyses

Results from sensitivity analyses are described in the eResults in Supplement 1, presented in eTables 4 to 6 in Supplement 1, and addressed in the eDiscussion in Supplement 1. Briefly, point estimates were generally similar after adjusting pollutants for each other, similar (and often higher) for participants who did not move house, and similar for complete cases, although CIs were often less precise.

## Discussion

In this longitudinal birth cohort study with a follow-up of approximately 25 years, participants exposed to higher PM_2.5_ during pregnancy and childhood subsequently experienced more psychotic experiences and (for pregnancy exposure only) depression. In contrast, higher noise pollution in childhood and adolescence were associated subsequently with more anxiety. These associations were not explained by numerous potential individual-, family-, and area-level confounders.

Our findings suggest an important role of early-life (including prenatal) exposure to air pollution in the development of youth mental health problems. Early-life exposure could be detrimental to mental health given the extensive brain development and epigenetic processes that occur in utero and during infancy.^13,15,49,50^ Air pollution exposure could also lead to restricted fetal growth^51^ and preterm birth,^52^ which are both risk factors for psychopathology. Notably, the point estimate for pregnancy PM_2.5_ and depression (10% elevated odds for every 0.72 μg/m^3^ increase) was considerably greater than a previous meta-analytic estimate based on exposure in adulthood (10% elevated odds for every 10 μg/m^3^ increase).^2^ These contrasting findings are in keeping with a particularly detrimental role of early-life air pollution exposure. However, our findings could also have arisen if early-life exposure data provide a proxy for cumulative exposure over a longer period, given that families often settle when children are young.

For noise pollution, evidence was strongest for childhood and adolescent exposure. Childhood and adolescent noise pollution exposure could increase anxiety by increasing stress and disrupting sleep, with high noise potentially leading to chronic physiological arousal and disruption to endocrinology.^53^ Noise pollution could also impact cognition,^54^ which could increase anxiety by impacting concentration during school years. It was interesting that noise pollution was associated with anxiety but not with psychotic experiences or depression. However, our measure of noise pollution estimated only decibels (ie, intensity) from road sources. Other qualities of noise, such as pitch, could be relevant to mental health.

### Limitations

We acknowledge several limitations. First, the causality of the findings is uncertain given that data were observational. Despite comprehensive covariate adjustment, residual confounding is inevitable given imperfect selection and measurement of covariates. The relatively large E values strengthened our confidence in the findings, but future studies should consider other methods to address confounding, such as quasi-experimental designs. Second, ALSPAC families are more affluent and less diverse than the UK population.^55^ The extent to which our findings generalize to other populations and locations is uncertain. Our findings likely generalize to cities and surrounds in other high-income countries, but may be less generalizable to urban settings in lower-income countries, which can have more extreme pollution concentrations.^56^ Third, modeled pollution data are subject to various sources of measurement error,^39^ particularly Berkson-like error whereby estimates are smoother (less variable) than reality, leading to less precise, although unbiased, exposure-outcome estimates.^57,58^ For instance, the 100 m^2^ resolution, although an improvement over many previous studies,^59,60,61^ would have masked hyperlocal variation (eg, differences between participants living on adjacent streets), to which NO_2_ is especially prone due to its short decay function.^62^ Additionally, the model estimated residential exposure, which would have masked variation due to behavior and time spent away from home. Finer-resolution data, including personal exposure estimates, would enable more precise exposure-outcome estimates, particularly for NO_2_. Fourth, we could not apply life-course models to investigate sensitive periods vs cumulative effects, as there was limited within-person variation in exposure over time. Larger data sets (eg, national registries) and quasi-experimental designs would be required to further tease out this question.

## Conclusions

The results of this cohort study provide novel evidence that early-life exposure to particulate matter is prospectively associated with the development of psychotic experiences and depression in youth. This study, which is among only a handful of longitudinal studies to investigate the association between noise pollution and mental health, also finds an association with anxiety. The findings suggest a degree of specificity in terms of pollutant-timing-outcome pathways. The opportunity for intervention is potentially enormous. However, although our this study addressed various biases affecting observational research, the causality of the findings remains uncertain. There is now a pressing need for further longitudinal research using more precise measures of air and noise pollution and for replication using quasi-experimental designs.

## Notes

Supplement 1. **eMethods.** Participants, pollution data, covariates, and multiple imputation **eResults.** Findings from sensitivity analyses **eDiscussion.** Interpretation of sensitivity analyses **eFigure 1.** Correlations between NO2, PM2.5, and noise pollution across pregnancy, childhood, and adolescence **eFigure 2.** Directed acyclic graph (DAG) **eTable 1.** Association of early-life noise pollution exposure with youth mental health problems, treating noise pollution as a categorical variable **eTable 2.** Comparison between e-value and covariate point estimates: pregnancy PM2.5 and psychotic experiences **eTable 3.** Comparison between e-value and covariate point estimates: adolescent noise pollution and anxiety **eTable 4.** Adjusting pollutants for one another: associations of early-life air and noise pollution exposure with youth mental health problems **eTable 5.** Restricting to non-movers (~30% of participants): associations of early-life air and noise pollution exposure with youth mental health problems **eTable 6.** Complete case analysis: associations of early-life air and noise pollution exposure with youth mental health problems

Supplement 2.
Data Sharing Statement
