# Supplementary material for: Air and Noise Pollution Exposure in Early Life and Mental Health From Adolescence to Young Adulthood
Source: JAMA Netw Open. 2024 May 28;7(5):e2412169. doi: 10.1001/jamanetworkopen.2024.12169 (PMC11134215; doi:10.1001/jamanetworkopen.2024.12169)
Supplement: Supplement 1. — eMethods. Participants, pollution data, covariates, and multiple imputation eResults. Findings from sensitivity analyses eDiscussion. Interpretation of sensitivity analyses eFigure 1. Correlations between NO2, PM2.5, and noise pollution across pregnancy, childhood, and adolescence eFigure 2. Directed acyclic graph (DAG) eTable 1. Association of early-life noise pollution exposure with youth mental health problems, treating noise pollution as a categorical variable eTable 2. Comparison between e-value and covariate point estimates: pregnancy PM2.5 and psychotic experiences eTable 3. Comparison between e-value and covariate point estimates: adolescent noise pollution and anxiety eTable 4. Adjusting pollutants for one another: associations of early-life air and noise pollution exposure with youth mental health problems eTable 5. Restricting to non-movers (~30% of participants): associations of early-life air and noise pollution exposure with youth mental health problems eTable 6. Complete case analysis: associations of early-life air and noise pollution exposure with youth mental health problems [file jamanetwopen-e2412169-s001.pdf]

Supplementary Online Content

Newbury JB, Heron J, Kirkbride JB, et al. Air and noise pollution exposure in early life and mental health from adolescence to young adulthood. *JAMA Netw Open*. 2024;7(5):e2412169. doi:10.1001/jamanetworkopen.2024.12169

eMethods. Participants, pollution data, covariates, and multiple imputation

eResults. Findings from sensitivity analyses

eDiscussion. Interpretation of sensitivity analyses

eFigure 1. Correlations between NO2, PM2.5, and noise pollution across pregnancy, childhood, and adolescence

eFigure 2. Directed acyclic graph (DAG)

eTable 1. Association of early-life noise pollution exposure with youth mental health problems, treating noise pollution as a categorical variable

eTable 2. Comparison between e-value and covariate point estimates: pregnancy PM2.5 and psychotic experiences

eTable 3. Comparison between e-value and covariate point estimates: adolescent noise pollution and anxiety

eTable 4. Adjusting pollutants for one another: associations of early-life air and noise pollution exposure with youth mental health problems

eTable 5. Restricting to non-movers (~30% of participants): associations of early-life air and noise pollution exposure with youth mental health problems

eTable 6. Complete case analysis: associations of early-life air and noise pollution exposure with youth mental health problems

eReferences. From eMethods

This supplementary material has been provided by the authors to give readers additional information about their work.

© 2024 Newbury JB et al. *JAMA Network Open*.

eMethods.

Participants

The Avon Longitudinal Study of Parents and Children (ALSPAC) is a UK birth cohort study.[1-3] Pregnant women resident in the county of Avon (in Southwest England) with an expected delivery date between 1st April 1991 and 31st December 1992 were approached to take part in the study, with 14,541 women initially recruited. When children were approximately 7 years of age, an attempt was made to bolster the initial sample to include additional children who met the original eligibility criteria, leading to a total sample size of 14,901 babies alive at 1 year of age. The catchment area includes the city of Bristol (population ~707,000 in 2023), small towns and villages; with a mix of urban, suburban and rural environments.[4] Study data from age 22 onwards were collected and managed using REDCap (Research Electronic Data Capture).[5] The study website contains details of all the data and a fully searchable data dictionary and variable search tool: http://www.bristol.ac.uk/alspac/researchers/our-data/. Ethical approval for the study was obtained from the ALSPAC Ethics and Law Committee and the Local Research Ethics Committees. Informed consent for the use of data collected via questionnaires and clinics was obtained from participants following the recommendations of the ALSPAC Ethics and Law Committee at the time.

Air pollution

Air pollutants included nitrogen dioxide (NO_2) and fine particulate matter with a diameter of <2.5 microns ($PM_{2.5}$). Both pollutants have well-established health impacts[6] and more recent associations with psychiatric disorders.[7] These air pollutants were estimated as part of the LifeCycle project[8] using the Effects of Low-Level Air Pollution: A Study in Europe (ELAPSE) model, which is described elsewhere.[9] Briefly, the ELAPSE model is a hybrid land-use regression model for Europe which derived concentrations of NO_2 and $PM_{2.5}$ in 2010. Inputs included the AirBase v8 dataset,[10] data from satellites and chemical transport models, and data on road use, land cover, and elevation. The model produces annualized estimates at $100m^2$ resolution, explaining 59% and 71% of measured spatial variability for NO_2 and $PM_{2.5}$, respectively.[9] Estimates were back-extrapolated to earlier years by conducting temporal adjustment using observed pollution data, and linked to all residential geocodes from pregnancy to age 12 for participants who had lived in the original ALSPAC catchment area[4] up to age 12 and had provided permission for geospatial linkage. Linkage was completed in 2020.

Covariates

Ethnicity of the child was reported by mothers during pregnancy. Most participants with mental health data were ethnically White (N=7,616, 95.8%). Due to the small numbers within other ethnicities, ethnicity was dichotomized as White versus Non-White (all other ethnicities).

Family psychiatric history was reported by mothers and fathers during pregnancy, and defined as the presence of schizophrenia, depression, drug addiction, alcoholism, or any other psychiatric problem in the mother, father, or the mother/father's biological mother or father. Over half of participants with mental health data (N=4,793, 60.8%) had at least one biological parent or grandparent with a history of psychiatric illness.

Maternal social class was reported by mothers during pregnancy based on occupation. Occupations were defined as professional (N=295, 4.1%), managerial and technical (N=2,302, 31.8%), skilled non-manual (N=3,068, 42.3%), skilled manual (N=264, 3.6%), partly skilled (N=1,096, 15.1%) and unskilled (N=223, 3.1%).

Maternal education was reported by mothers when babies were around 8 months. Education levels included Certificate of Secondary Education (CSE) (N=1,152, 14.2%), vocational qualifications (N=730, 9.0%), O level (N=2,850, 35.2%), A level (N=2,087, 25.8%), and degree (N=1,274, 15.7%).

Home ownership was reported by mothers during pregnancy and defined according to mortgaged/owned (N=6,670, 81.6%) versus rented (N=1,506, 18.4%).

Population density was derived from 1991 census data (or 2001 census data for adolescence exposure models) and defined as persons per hectare.[11] Population density data were linked to address geocodes using non-identifying linkage methods. The time-period of population density was chosen to correspond with the timing of pollution exposure (i.e., pregnancy, age 1 (for pollution exposure in childhood), and age 10 (for pollution exposure in adolescence).

© 2024 Newbury JB et al. *JAMA Network Open*.

Area-level deprivation was based on the 2000 Index of Multiple Deprivation (IMD).[12] The 2000 IMD ranked the 8,414 England wards according to deprivation markers in the domains of income, employment, health, education, housing, access, and child poverty. These ranks were then placed into quintiles, with the top quintile (5) representing the highest deprivation. Again, data were linked to address geocodes via non-identifying methods. Again, the time-period of area-level deprivation corresponded with the timing of pollution exposure.

Social fragmentation was based on z-scoring and summing the following census data: percentage of people who moved in the last year; percentage of unmarried people aged 16 and over; percentage of single person households; and percentage of privately rented households.[11] For pregnancy and childhood models, 1991 census data were used. For adolescence models, 2001 census data were used. Again, data were linked to address geocodes via non-identifying methods. Again, the time-period of social fragmentation corresponded with the timing of pollution exposure.

Greenspace was assessed based on the Normalized Difference Vegetation Index (NDVI), derived from satellite imagery, described in detail previously.[13] Data were derived for all ALSPAC residents living in Southwest England and Wales. The present study uses estimates of greenness within a 300-meter buffer zone around residential addresses, which is commonly used by the World Health Organization as an accessibility threshold.[14] Again, data were linked to address geocodes via non-identifying methods. Again, the time-period of greenspace corresponded with the timing of pollution exposure.

Multiple imputation via chained equations

We imputed missing covariate data for participants with complete pollution and mental health data. To maximise the potential size of imputed datasets, imputation models were conducted separately for the three mental health outcomes; for air and noise pollution; and for different exposure periods. Multiple imputation was performed within Stata v17.0 ("mi impute chained"). Imputed variables included ethnicity, family psychiatric history, maternal social class, maternal education, home ownership, population density, area-level deprivation, social fragmentation, and greenspace, with each variable specified as a continuous, ordinal, count, or binary variable, as appropriate. We selected the following pregnancy variables as auxiliary variables: maternal marital status, crowding index, and neighbourhood quality index. We imputed 5 datasets using a random seed of 1234. Sample sizes of imputed datasets ranged from N=2,962 (adolescent noise pollution and psychotic experiences) to N=6,180 (pregnancy air pollution and anxiety).

© 2024 Newbury JB et al. *JAMA Network Open*.

eResults

Sensitivity analysis

In the first sensitivity analysis (adjustment for other pollutants), the associations of pregnancy and childhood $PM_{2.5}$ with psychotic experiences were robust to adjustment for NO_2 and noise. The point estimate for the association of pregnancy $PM_{2.5}$ with depression was similar to the original association, though confidence intervals crossed the null. Likewise, the associations of childhood and adolescence noise pollution with anxiety were similar to the original associations, though confidence intervals crossed the null (eTable 4).

In the second sensitivity analysis, analyses were restricted to participants who did not move house between pregnancy and age 12 [29.8% of sample] to keep pollution levels as consistent over time as possible. In general, point estimates were similar in magnitude to the original association, though confidence intervals were less precise and usually crossed the null, potentially due to the smaller sample size. However, the association of pregnancy $PM_{2.5}$ with depression was substantially attenuated among the non-mover subsample (aOR=0.97, 95 CI=0.84-1.13, p=0.72). Several associations increased in magnitude for non-movers, including the association of adolescent $PM_{2.5}$ and NO_2 exposure with psychotic experiences; and the association of noise pollution at all ages with all mental health outcomes (eTable 5).

In the third sensitivity analysis (complete case analyses), point estimates were generally similar to those from multiply imputed models, though confidence intervals were often less precise, again, potentially due to the smaller sample size (eTable 6).

© 2024 Newbury JB et al. *JAMA Network Open*.

eDiscussion.

Sensitivity analysis

Results from the first sensitivity analysis, in terms of the magnitude of effect sizes, suggest that the association between $PM_{2.5}$ and psychotic experiences was not confounded by correlations with NO_2 or noise pollution. Similarly, the associations of pregnancy $PM_{2.5}$ with depression; and of childhood and adolescence noise pollution with anxiety, were not explained by correlations with the other pollutants. However, confidence intervals were often wider and included the null. This could potentially be explained by the high correlations between pollutants, which could lead to multi-collinearity when analysed simultaneously. Furthermore, it remains possible that other correlated pollutants contributed to the observed associations.

Results from the second sensitivity analysis are in keeping with a role of cumulative exposure over a longer duration. For instance, the magnitude of associations of adolescent NO_2 and $PM_{2.5}$ with psychotic experiences was stronger when restricted to participants who did not move house from pregnancy to age 12, and who therefore had more cumulative exposure over a longer duration. Similarly, the pattern of findings for noise pollution is in keeping with a compounding of exposure over time (i.e., cumulative effects), given that across all outcomes, point estimates were larger in magnitude for participants who did not move house, and therefore had similar noise pollution exposure over time.

However, we interpret the results from these sensitivity analyses with caution, given that the high correlations between pollutants (eFigure 1) could introduce multi-collinearity when analysed simultaneously; although the variance inflation factors (VIF) for the co-pollutant models were VIF=1.4-1.6, suggesting that multi-collinearity was not very high. Additionally, results for non-movers should be interpreted with caution given that this subsample was a minority (29.8%) and may not be representative of the full sample.

© 2024 Newbury JB et al. *JAMA Network Open*.

eFigure 1. Correlations between NO_2, $PM_{2.5}$, and noise pollution across pregnancy, childhood, and adolescence

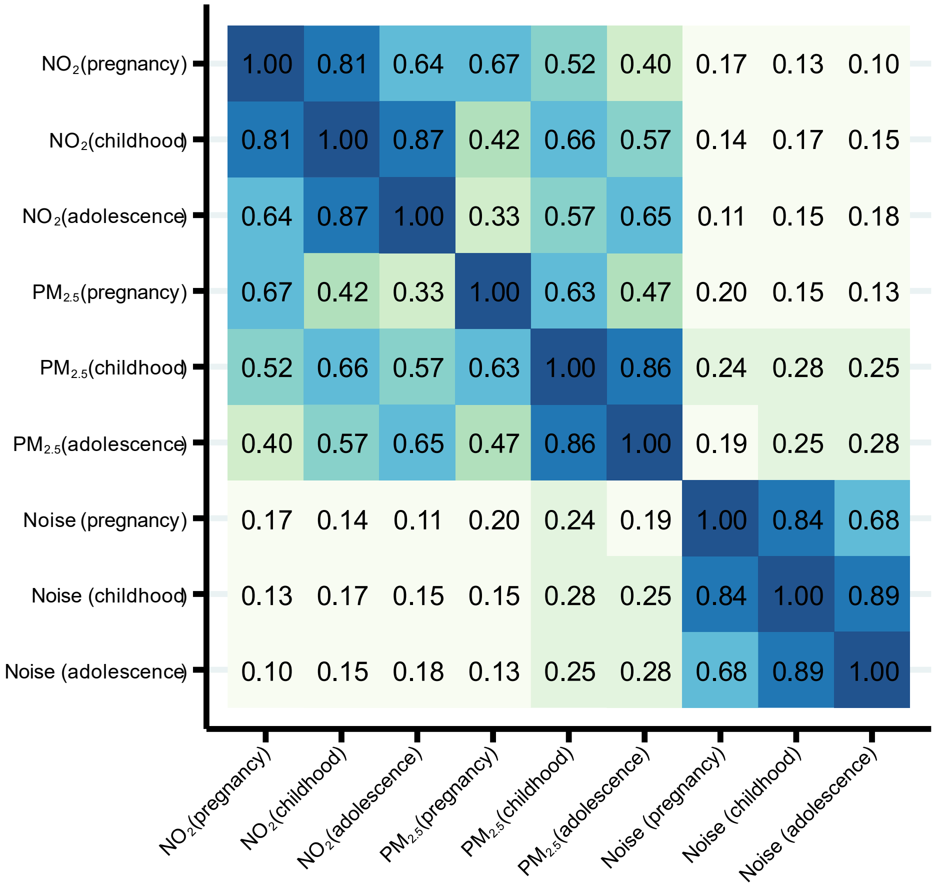

Note: NO_2=nitrogen dioxide; $PM_{2.5}$=particulate matter under 2.5 microns; calculated using Polychoric correlations; all p's<0.001.

© 2024 Newbury JB et al. *JAMA Network Open*.

eFigure 2. Directed acyclic graph (DAG)

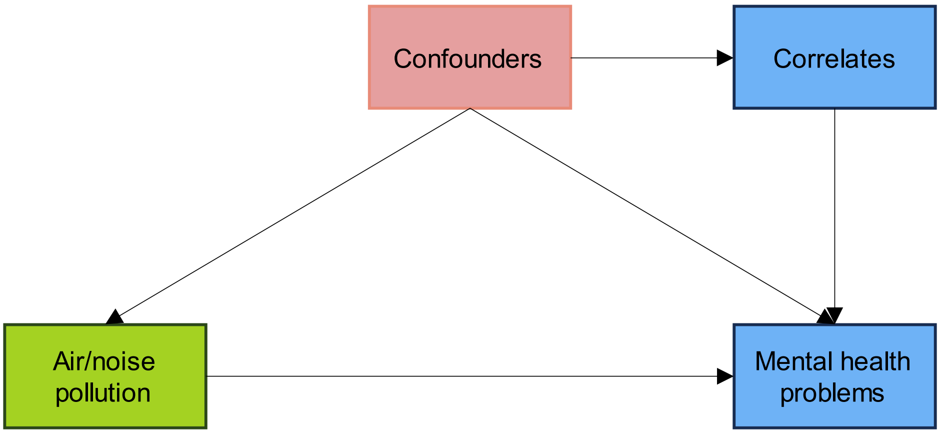

Note: Directed acyclic graph (DAG) is adapted from dagitty.net. Confounders included: ethnicity, family psychiatric history, maternal social class, maternal education, housing tenure, population density, neighborhood deprivation, social fragmentation, and greenspace. Other correlates were considered but not included as confounders given results of the DAG (e.g., they were mediators and/or could not cause exposure to outdoor air/noise pollution): housing quality, nutrition, household crowding, maternal smoking, preterm birth, and restricted fetal growth.

© 2024 Newbury JB et al. *JAMA Network Open*.

eTable 1. Association of early-life noise pollution exposure with youth mental health problems, treating noise pollution as a categorical variable

Outcome	Pregnancy exposure			Childhood exposure			Adolescence exposure		
Noise pollution level	OR (95% CI)	P-value	LR X^2 (P-value)	OR (95% CI)	P-value	LR X^2 (P-value)	OR (95% CI)	P-value	LR X^2 (P-value)
Psychotic experiences									
Low-medium noise	Reference	-		Reference	-		Reference	-	
High noise	0.95 (0.78-1.15)	0.59		0.93 (0.76-1.13)	0.44		0.85 (0.69-1.05)	0.14	
Very high noise	1.10 (0.86-1.41)	0.44	0.03 (0.86)	1.04 (0.82-1.32)	0.77	1.66 (0.20)	1.03 (0.79-1.34)	0.82	0.49 (0.49)
Depression									
Low-medium noise	Reference	-		Reference	-		Reference	-	
High noise	0.88 (0.69-1.12)	0.30		1.01 (0.80-1.28)	0.94		0.90 (0.68-1.17)	0.42	
Very high noise	1.07 (0.81-1.41)	0.62	1.29 (0.26)	1.27 (0.93-1.72)	0.13	1.21 (0.27)	1.12 (0.83-1.53)	0.46	0.03 (0.86)
Anxiety									
Low-medium noise	Reference	-		Reference	-		Reference	-	
High noise	1.04 (0.82-1.31)	0.76		1.17 (0.92-1.50)	0.20		1.17 (0.87-1.58)	0.31	
Very high noise	1.10 (0.83-1.46)	0.51	0.03 (0.86)	1.42 (1.05-1.90)	0.02	0.00 (0.98)	1.48 (1.05-2.11)	0.03	0.5 (0.48)

Note: CI=confidence interval; LR X^2=likelihood-ratio Chi-square test; All models are adjusted for individual-, family- and area-level covariates.

© 2024 Newbury JB et al. *JAMA Network Open*.

eTable 2. Comparison between e-value and covariate point estimates: pregnancy PM2.5 and psychotic experiences

E-values/Covariates	Association with pregnancy $PM_{2.5}$			Association with psychotic experiences		
	OR [a]	LCL / 95% CI [b]	P-value	OR	LCL / 95% CI [b]	P-value
E-value	1.46	1.24	-	1.46	1.24	-
Ethnicity	1.05	0.96-1.15	0.28	1.20	0.88-1.63	0.25
Family psychiatric history	0.99	0.95-1.03	0.62	1.15	0.98-1.34	0.08
House tenure	0.95	0.89-1.02	0.16	1.34	1.11-1.61	0.002
Maternal social class [c]						
Professional	Reference	-	-	Reference	-	-
Managerial and technical	1.11	0.99-1.24	0.08	0.88	0.61-1.27	0.48
Skilled non-manual	1.08	0.96-1.21	0.20	0.90	0.62-1.30	0.56
Skilled manual	0.99	0.85-1.14	0.86	0.93	0.54-1.58	0.78
Partly skilled	1.07	0.94-1.21	0.34	0.83	0.55-1.24	0.36
Unskilled	0.99	0.84-1.16	0.89	0.88	0.48-1.59	0.66
Maternal education						
Degree	Reference	-	-	Reference	-	-
A level	1.05	0.98-1.13	0.15	1.13	0.89-1.44	0.32
O level	1.15	1.07-1.24	<0.001	1.15	0.90-1.47	0.26
Vocational	1.14	1.04-1.25	0.004	1.20	0.90-1.62	0.21
CSE	1.12	1.03-1.23	0.01	1.24	0.92-1.68	0.16
Population density	1.00	1.00-1.00	0.47	1.00	1.00-1.00	0.93
Area-level deprivation						
1 – Least deprived	Reference	-	-	Reference	-	-
2	1.12	0.96-1.30	0.15	0.99	0.75-1.32	0.96
3	1.07	0.96-1.20	0.23	1.01	0.79-1.30	0.94
4	1.07	0.96-1.19	0.23	0.92	0.71-1.21	0.56
5 – Most deprived	1.11	1.00-1.24	0.06	1.07	0.83-1.37	0.61
Social fragmentation	1.00	0.98-1.01	0.82	1.03	1.00-1.06	0.03
Greenspace	0.04	0.02-0.08	<0.001	0.92	0.35-2.46	0.88

© 2024 Newbury JB et al. *JAMA Network Open*.

Note: [a]linear regression was used to analyse the association of covariates with $PM_{2.5}$, a continuous variable. Beta coefficients from linear regression models were exponentiated to aid comparison with e-value ORs; [b]E-values do not include p-values or upper confidence limits; [c]based on maternal occupation; CI=confidence interval; CSE=certificate of secondary education; LCL=lower confidence limit; OR=odds ratio; $PM_{2.5}$=particulate matter <2.5 microns.

© 2024 Newbury JB et al. *JAMA Network Open*.

eTable 3. Comparison between e-value and covariate point estimates: adolescent noise pollution and anxiety

**E-values/Covariates**	**Association with adolescent noise pollution**			**Association with anxiety**		
	OR [a]	LCL / 95% CI [b]	P-value	OR	LCL / 95% CI [b]	P-value
E-value	1.74	1.16	-	1.74	1.16	-
Ethnicity	1.02	0.72-1.46	0.90	0.72	0.37-1.41	0.33
Family psychiatric history	0.95	0.83-1.10	0.50	1.05	0.80-1.38	0.71
House tenure	1.09	0.91-1.32	0.35	1.50	1.10-2.05	0.01
Maternal social class [c]						
Professional	Reference	-	-	Reference	-	-
Managerial and technical	0.88	0.61-1.28	0.51	0.78	0.43-1.41	0.41
Skilled non-manual	1.02	0.68-1.53	0.92	0.70	0.38-1.30	0.26
Skilled manual	1.28	0.73-2.25	0.38	0.43	0.16-1.15	0.09
Partly skilled	0.80	0.50-1.27	0.33	0.73	0.38-1.41	0.35
Unskilled	0.93	0.52-1.65	0.81	0.52	0.17-1.55	0.24
Maternal education						
Degree	Reference	-	-	Reference	-	-
A level	1.40	1.09-1.81	0.009	1.11	0.75-1.64	0.60
O level	1.48	1.14-1.93	0.003	0.93	0.62-1.41	0.75
Vocational	1.36	0.98-1.87	0.06	0.94	0.55-1.60	0.82
CSE	1.65	1.19-2.28	0.003	0.69	0.42-1.16	0.16
Population density	0.99	0.98-0.99	<0.001	1.00	1.00-1.00	0.44
Area-level deprivation						
1 – Least deprived	Reference	-	-	Reference	-	-
2	0.99	0.70-1.40	0.95	0.89	0.57-1.39	0.61
3	1.34	0.96-1.87	0.09	0.92	0.64-1.32	0.65
4	1.04	0.74-1.46	0.81	1.28	0.92-1.77	0.14
5 – Most deprived	1.11	0.81-1.53	0.51	1.04	0.72-1.50	0.85
Social fragmentation	1.01	0.98-1.05	0.57	1.02	0.98-1.06	0.42
Greenspace	0.30	0.10-0.90	0.03	1.29	0.28-6.00	0.75

© 2024 Newbury JB et al. *JAMA Network Open*.

Note: [a]E-values do not include p-values or upper confidence limits; [b]based on maternal occupation; CSE=certificate of secondary education; CI=confidence interval; LCL=lower confidence limit; OR=odds ratio.

© 2024 Newbury JB et al. *JAMA Network Open*.

eTable 4. Adjusting pollutants for one another: associations of early-life air and noise pollution exposure with youth mental health problems

Outcome	Pregnancy exposure		Childhood exposure		Adolescence exposure	
Pollutant	OR (95% CI)	P-value	OR (95% CI)	P-value	OR (95% CI)	P-value
Psychotic experiences						
NO_2	0.97 (0.88-1.09)	0.64	0.92 (0.82-1.02)	0.11	0.92 (0.79-1.05)	0.22
$PM_{2.5}$	1.11 (1.04-1.19)	0.003	1.12 (1.02-1.22)	0.02	1.10 (0.98-1.23)	0.12
Noise	1.05 (0.89-1.25)	0.53	1.01 (0.89-1.14)	0.89	0.98 (0.85-1.14)	0.82
Depression						
NO_2	1.05 (0.92-1.20)	0.47	1.08 (0.94-1.23)	0.29	1.09 (0.93-1.27)	0.30
$PM_{2.5}$	1.08 (1.00-1.17)	0.06	1.03 (0.94-1.14)	0.51	0.95 (0.85-1.07)	0.41
Noise	1.03 (0.87-1.21)	0.72	1.12 (0.96-1.29)	0.14	1.09 (0.91-1.31)	0.32
Anxiety						
NO_2	1.07 (0.93-1.23)	0.36	1.07 (0.93-1.23)	0.35	0.97 (0.81-1.16)	0.71
$PM_{2.5}$	1.01 (0.92-1.10)	0.88	0.99 (0.89-1.09)	0.78	0.96 (0.85-1.08)	0.49
Noise	1.00 (0.88-1.14)	0.99	1.25 (1.05-1.49)	0.02	1.20 (1.02-1.42)	0.03

Note: CI=confidence interval; NO_2=nitrogen dioxide; OR=odds ratio; $PM_{2.5}$=particulate matter under 2.5 microns; Each pollutant is adjusted for the other pollutants (e.g., NO_2 is adjusted for $PM_{2.5}$ and noise pollution); All models are adjusted for individual-, family- and area-level covariates.

© 2024 Newbury JB et al. *JAMA Network Open*.

eTable 5. Restricting to non-movers (~30% of participants): associations of early-life air and noise pollution exposure with youth mental health problems

Outcome	Pregnancy exposure			Childhood exposure			Adolescence exposure		
Pollutant	N	OR (95% CI)	P-value	N	OR (95% CI)	P-value	N	OR (95% CI)	P-value
Psychotic experiences									
NO_2	1,359	1.02 (0.81-1.28)	0.85	1,327	0.99 (0.77-1.28)	0.96	1,257	1.08 (0.83-1.41)	0.56
$PM_{2.5}$	1,359	1.11 (0.98-1.26)	0.11	1,327	1.13 (0.96-1.32)	0.14	1,257	1.12 (0.94-1.32)	0.21
Noise	887	1.20 (0.92-1.58)	0.18	854	1.16 (0.87-1.54)	0.32	810	1.20 (0.91-1.59)	0.19
Depression									
NO_2	1,540	0.94 (0.76-1.18)	0.61	1,503	0.98 (0.76-1.27)	0.88	1,396	1.07 (0.80-1.44)	0.65
$PM_{2.5}$	1,540	0.97 (0.84-1.13)	0.72	1,503	0.95 (0.80-1.14)	0.61	1,396	0.99 (0.81-1.22)	0.96
Noise	1,003	1.61 (1.13-2.28)	0.008	966	1.53 (1.07-2.19)	0.02	896	1.49 (1.05-2.12)	0.03
Anxiety									
NO_2	1,556	1.05 (0.82-1.34)	0.69	1,518	1.05 (0.79-1.38)	0.74	1,409	0.94 (0.65-1.35)	0.74
$PM_{2.5}$	1,556	0.97 (0.83-1.13)	0.67	1,518	0.94 (0.79-1.13)	0.51	1,409	0.87 (0.70-1.08)	0.22
Noise	1,012	1.30 (0.95-1.77)	0.10	974	1.31 (0.96-1.80)	0.09	903	1.38 (0.98-1.95)	0.06

Note: CI=confidence interval; NO_2=nitrogen dioxide; OR=odds ratio; $PM_{2.5}$=particulate matter under 2.5 microns; The analytic sample is restricted to participants who did not move house between pregnancy and age 12 (29.8% of sample); All models are adjusted for individual-, family- and area-level covariates.

© 2024 Newbury JB et al. *JAMA Network Open*.

eTable 6. Complete case analysis: associations of early-life air and noise pollution exposure with youth mental health problems

Outcome	Pregnancy exposure			Childhood exposure			Adolescence exposure		
Pollutant	N	OR (95% CI)	P-value	N	OR (95% CI)	P-value	N	OR (95% CI)	P-value
Psychotic experiences									
NO_2	3,127	1.00 (0.87-1.14)	0.97	4,865	0.98 (0.88-1.09)	0.72	3,849	0.94 (0.81-1.09)	0.40
$PM_{2.5}$	3,127	1.12 (1.03-1.22)	0.01	4,865	1.10 (1.01-1.21)	0.02	3,849	1.07 (0.95-1.21)	0.28
Noise	2,117	1.06 (0.90-1.25)	0.47	3,344	0.98 (0.86-1.13)	0.80	2,368	0.91 (0.78-1.06)	0.22
Depression									
NO_2	3,345	1.08 (0.93-1.26)	0.32	5,222	1.09 (0.95-1.24)	0.21	4,077	1.19 (1.01-1.41)	0.04
$PM_{2.5}$	3,345	1.10 (0.99-1.22)	0.08	5,222	1.09 (0.99-1.19)	0.072	4,077	1.01 (0.90-1.13)	0.91
Noise	2,244	1.03 (0.84-1.26)	0.76	3,567	1.15 (0.97-1.36)	0.12	2,499	1.10 (0.92-1.32)	0.30
Anxiety									
NO_2	3,363	1.03 (0.85-1.25)	0.73	5,253	1.07 (0.93-1.23)	0.35	4,101	1.05 (0.88-1.25)	0.61
$PM_{2.5}$	3,363	1.01 (0.90-1.14)	0.83	5,253	1.00 (0.90-1.11)	0.99	4,101	0.99 (0.87-1.11)	0.81
Noise	2,255	1.01 (0.83-1.24)	0.90	3,587	1.24 (1.05-1.47)	0.01	2,513	1.19 (0.98-1.44)	0.09

Note: CI=confidence interval; NO_2=nitrogen dioxide; OR=odds ratio; $PM_{2.5}$=particulate matter under 2.5 microns; Analyses were restricted to participants with complete covariate data (i.e., conducted without multiple imputation); N=2,117-5,253; All models are adjusted for individual-, family- and area-level covariates.

© 2024 Newbury JB et al. *JAMA Network Open*.

eReferences. From eMethods

1. Boyd A, Golding J, Macleod J, et al. Cohort profile: The ‘Children of the 90s’—the index offspring of the Avon Longitudinal Study of Parents and Children. *Int J Epidemiol.* 2013; 42(1): 111-127. doi:doi.org/10.1093/ije/dys064

2. Fraser A, Macdonald-Wallis C, Tilling K, et al. Cohort profile: the Avon Longitudinal Study of Parents and Children: ALSPAC mothers cohort. *Int J Epidemiol.* 2013; 42(1): 97-110. doi:10.1093/ije/dys066

3. Northstone K, Lewcock M, Groom A, et al. The Avon Longitudinal Study of Parents and Children (ALSPAC): an update on the enrolled sample of index children in 2019. *Wellcome Open Res.* 2019; 4. doi:10.12688/wellcomeopenres.15132.1

4. Boyd A, Thomas R, Hansell AL, et al. Data Resource Profile: The ALSPAC birth cohort as a platform to study the relationship of environment and health and social factors. *Int J Epidemiol.* 2019; 48(4): 1038-1039k. doi:10.1093/ije/dyz063

5. Harris PA, Taylor R, Thielke R, et al. Research electronic data capture (REDCap) - a metadata-driven methodology and workflow process for providing translational research informatics support. *J Biomed Inform.* 2009; 42(2): 377-381. doi:10.1016/j.jbi.2008.08.010

6. World Health Organization. Ambient air pollution: A global assessment of exposure and burden of disease. http://www.who.int/phe/publications/air-pollution-global-assessment/en/. Published 2016. World Health Organization.

7. Newbury JB, Stewart R, Fisher HL, et al. Association between air pollution exposure and mental health service use among individuals with first presentations of psychotic and mood disorders: retrospective cohort study. *Br J Psychiatry.* 2021; 219: 678-685. doi:10.1192/bjp.2021.119

8. de Castro Pascual M, Fossati S, Nieuwenhuijsen M, Vrijheid M. Protocol for integrated urban environment stressors generation in LifeCycle (WP3–Task 3.3). Rotterdam, The Netherlands: Erasmus MC, 2021.

9. De Hoogh K, Chen J, Gulliver J, et al. Spatial $PM_{2.5}$, NO_2, O_3 and BC models for Western Europe–Evaluation of spatiotemporal stability. *Environ Int.* 2018; 120: 81-92. doi:10.1016/j.envint.2018.07.036

10. European Environment Agency. Airbase - The European Air Quality Database, Version 8. 2015.

11. Solmi F, Lewis G, Zammit S, Kirkbride JB. Neighborhood characteristics at birth and positive and negative psychotic symptoms in adolescence: Findings from the ALSPAC birth cohort. *Schizophr Bull.* 2020; 46(3): 581-591. doi:10.1093/schbul/sbz049

12. DETR. Indices of Deprivation 2000. London: Department of the Environment, Transport and the Regions, 2000.

13. Fuertes E, Markevych I, Thomas R, et al. Residential greenspace and lung function up to 24 years of age: The ALSPAC birth cohort. *Environ Int.* 2020; 140: 105749. doi:10.1016/j.envint.2020.105749

14. Annerstedt Van Den Bosch M, Mudu P, Uscila V, et al. Development of an urban green space indicator and the public health rationale. *Scandinavian Journal of Public Health.* 2016; 44(2): 159-167. doi:10.1177/1403494815615444

© 2024 Newbury JB et al. *JAMA Network Open.*
