# Supplementary material for: Air and Noise Pollution Exposure in Early Life and Mental Health From Adolescence to Young Adulthood
Source: JAMA Netw Open. 2024 May 28;7(5):e2412169. doi: 10.1001/jamanetworkopen.2024.12169 (PMC11134215; doi:10.1001/jamanetworkopen.2024.12169)
Supplement: Supplement 2. — Data Sharing Statement [file jamanetwopen-e2412169-s002.pdf]

Data Sharing Statement

Newbury. Air and Noise Pollution Exposure in Early Life and Mental Health From Adolescence to Young Adulthood. *JAMA Netw Open*. Published May 28, 2024. doi:10.1001/jamanetworkopen.2024.12169

Data
Data available: No

Additional Information
Explanation for why data not available: The data are publicly available to view and request from the ALSPAC team, but cannot be made available by the authors. Upon manuscript acceptance, statistical code will be shared.
